# Ukrainian refugee crisis: the experience of the Roman Local Health Authority “ASL Roma 1”: Andriy Melnyk

**DOI:** 10.1093/eurpub/ckac131.029

**Published:** 2022-10-25

**Authors:** A Melnyk, LC Barone, S Messina, A Grossi, M Kohut, F Cascini, G Damiani, P Parente, M Goletti

**Affiliations:** 1 Hygiene, Life Sciences and Public Health, Catholic University of the Sacred Heart, Rome, Italy; 2 Women, Children and Public Health Sciences, Gemelli University Hospital, Rome, Italy; 3 Ukraine Emergency Coordination Unit, Italian Local Health Authority, ASL ROMA 1, Rome, Italy; 4 Health Management Directorate, Italian Local Health Authority, ASL ROMA 1, Rome, Italy

## Abstract

**Problem:**

On February 24th, 2022, Ukraine was invaded by Russian forces, forcing many Ukrainians to flee from their homes as refugees. More than 55,000 Ukrainians have since arrived on Italian territory. In response to the humanitarian crisis, the Roman Local Health Authority “ASL Roma 1” provided socio-sanitary assistance through first reception centers to more than 7700 refugees, prioritizing people with high social vulnerability. Ukraine’s vaccine hesitancy and different epidemiological landscape represented a major hurdle to be overcome.

**Practice:**

ASL Roma 1’s practice served to ensure infectious diseases prevention and control, as well as continuity of care for non-communicable diseases and mental health issues. It consisted of repurposing resources, such as COVID-19 Hubs and their personnel, stipulating Public-Private Partnerships and collaborations with the local Ukrainian community, massive training, creating a centralized multidisciplinary team (with Ukrainian members) and a dedicated database/IT system.

**Results:**

ASL Roma 1 empowered local Ukrainian communities by providing equipment, medical and administrative staff and socio-sanitary assistance. Ukrainian volunteers helped bridge the cultural gap for essential service provision, such as COVID-19 screening, enrolment in the NHS, health and social orientation, vaccinations and a tailored care pathway. Thus, more than 7700 refugees were assisted, with 1830 COVID-19 vaccinations administered and 170 in critical conditions promptly receiving specialized care.

**Lessons:**

The multidisciplinary and cross-cultural interaction between doctors, nurses, cultural mediators, social workers, and other key actors was essential in ensuring a holistic care pathway. Services catered to Ukrainian refugees need complete integration between primary and centralized care. Flexibility and resilience are fundamental to foster an ecosystem of innovation and optimization of healthcare provision on all levels, from local to supranational.

**Key messages:**

• The multidisciplinary and cross-cultural interaction between all medical and non-medical key actors is essential in ensuring a holistic care pathway and complete social integration of asylum seekers.

• Health system flexibility, resilience and an ecosystem of innovation and optimization of healthcare provision on all levels are fundamental components of preparedness for future refugee crises.

